# Colesevelam‐induced hypoglycaemia in a patient with type 1 diabetes mellitus

**DOI:** 10.1002/ccr3.4830

**Published:** 2021-10-15

**Authors:** Panagiotis Pavlou, Vaios Koutroukas, Catherine Lissett, Jamie C. Smith

**Affiliations:** ^1^ Diabetes and Endocrinology South Devon Healthcare NHS Foundation Trust Hengrave House Torbay Hospital Torquay UK

**Keywords:** colesevelam, diabetes, hypoglycemia, type 1 diabetes

## Abstract

Colesevelam possesses antidiabetic properties, which could potentiate sulphonylurea or insulin‐induced hypoglycemia; clinically significant hypoglycemia, as a side effect to bile acid sequestrants, may be under‐recognized in clinical practice.

## Graphical abstract

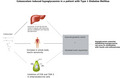

## 1 INTRODUCTION

We report the case of a 47‐year‐old female patient with type 1 diabetes mellitus receiving insulin pump therapy, who experienced severe hypoglycemia when commenced on colesevelam for bile acid malabsorption; symptoms resolved once the medication was discontinued, highlighting the potential of bile acid sequestrants for hypoglycemia. Action mechanisms are discussed.

Colesevelam is known to reduce LDL cholesterol and glucose.^1^ Colesevelam's glucose‐lowering effect, although not fully understood,^2^ is thought to be mediated by altering glucose metabolism in the intestine and liver.^3^ Although instances of hypoglycemia linked to colesevelam have been reported,^4^ there is no mention of this potential side‐effect in the summary of product characteristics (SmPC) for either colesevelam or its sister drug, cholestyramine.

We report the case of a 47‐year‐old female patient with type 1 diabetes mellitus (T1DM) receiving insulin pump therapy, who was started on colesevelam for bile acid malabsorption. While taking the drug, she experienced a number of severe hypoglycemic episodes; symptoms resolved once the medication was discontinued.

The case highlights that caution should be exercised when prescribing colesevelam or other bile acid sequestrants (BAS) in people with type 1 diabetes; mechanisms of action are further discussed.

## 2 CASE HISTORY

A 47‐year‐old female patient with type 1 diabetes mellitus (T1DM) receiving insulin pump therapy attended the diabetes outpatient clinic for annual follow‐up. She had T1DM of 22 years duration with reasonably good glycemic control (HbA1c of 62 mmol/mol); she had been commenced on insulin pump therapy 2 years before because of severe hypoglycemia on a daily basis, despite optimized specialist diabetes care and education. Following the initiation of pump therapy, blood glucose levels stabilized, with hypoglycemia occurring approximately once monthly.

Past medical history included bile acid (BA) malabsorption of unknown etiology, background diabetic retinopathy, and hypercholesterolemia. She was of slim build; body mass index (BMI) was 21.1 kg/m^2^.

Colesevelam 625 mg (2–3 times daily) was initiated by her gastroenterologist because of nausea, belching, and diarrhea, as a 10‐day trial in the hope of symptomatic relief. However, 3 days post‐commencement she started experiencing recurrent and severe hypoglycemia (as low as 1.8 mmol/L).

On the 7^th^ day, she discontinued the medication because of concerns over hypoglycemia, despite an improvement in her gastrointestinal symptoms. Its cessation led to resolution of her hypoglycemic symptoms; she denied any changes to her insulin regime, diet, weight, or physical activity during this period.

Because of colesevelam's effectiveness in relieving gastrointestinal symptoms, she was keen to restart it, agreeing to colesevelam, and insulin dose adjustments. On endocrinological advice, she reduced both prandial and basal insulin doses by 30%, while colesevelam was cautiously re‐introduced at a dose of 625 mg with meals, 7 days following the initial 7‐day trial.

## 3 INVESTIGATIONS

**FIGURE 1:**
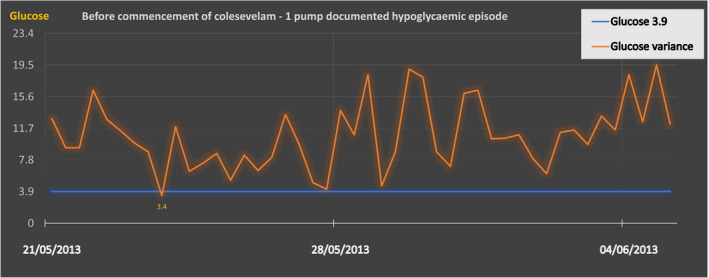
Before commencement of colesevelam ‐ 1 pump documented hypoglycaemic episode

**FIGURE 2:**
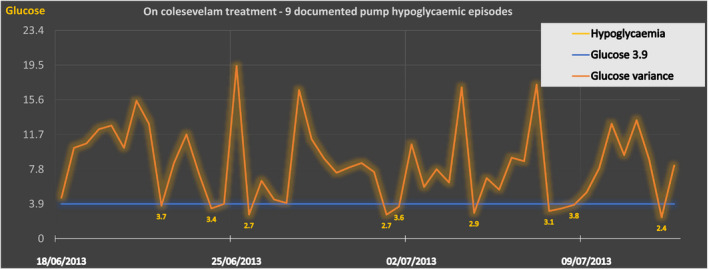
On colesevelam treatment ‐ 9 documented hypoglycaemic episodes

According to the pump‐generated reports (Figure 1), prior to colesevelam initiation, only 1% of total capillary blood glucose readings were within the hypoglycemic range (<4 mmol/L); this increased to 7% post‐recommencement. The frequency of hypoglycemia increased in both fasting and postprandial periods. Her average total daily insulin dosage decreased from 45.2 IU (0.72 IU/kg) to 29.9 IU (0.46 IU/kg). HbA1c was lowered from 62 to 58 mmol/mol. However, due to constipation and recurrence of hypoglycemia, it was permanently discontinued, following the second four‐week trial (Figure 2).

## 4 OUTCOME

**FIGURE 3:**
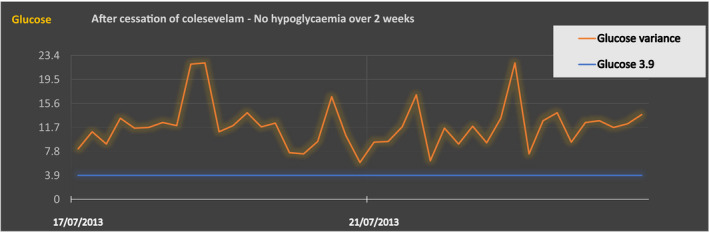
After cessation of colesevelam ‐ No hypoglycaemia over 2 weeks

Once colesevelam stopped, hypoglycemic episodes resolved (Figure 3). The side effects experienced by our patient were reported to the MHRA (Medicines and Healthcare Products Regulatory Agency) through the Yellow Card reporting scheme.

## 5 DISCUSSION

Colesevelam is known to reduce LDL cholesterol and glucose.^1^ Colesevelam's glucose‐lowering effect, although not fully understood,^2^ is thought to be mediated by altering glucose metabolism in the intestine and liver.^3^

Colesevelam is a BA sequestrant (BAS) binding to bile acids (BA) forming the BAS‐BA complex,^4^ which induces glucagon‐like peptide‐1 (GLP‐1) production by the enteroendocrine L cells in the intestine, potentiating the insulin effect. The effect could be possibly mediated through the inhibition of farnesoid X receptor (FXR) and G protein‐coupled bile acid receptor 1 (TGR5).^3^ FXR is a nuclear receptor mainly expressed in the liver, the intestine (possibly in L‐cells, too), while TGR5 can be found in the small and large intestine, gall bladder, and ileum.^3^ In animal studies and human intestinal biopsies, it has been found that FXR activation decreases proglucagon mRNA levels, inhibits glucose‐induced proglucagon expression, and decreases glycolysis and glucose‐induced GLP‐1 secretion. Moreover, FXR deficiency increases proglucagon mRNA and GLP‐1 levels, improving glycemia through the GLP‐1R pathway: overall, BA sequestrants improve glycemia and GLP‐1 production via FXR,^5^ although the effect is rather modest when compared to dipeptidyl peptidase‐4 inhibitors.^2^

Another mechanism proposed, that may contribute to glucose lowering, involves the cholesterol‐linked post‐transcriptional gene regulation in liver: colesevelam induces the expression of the miR‐96/182/183 cluster of miRNAs in the hepatic cells, likely through increased sterol regulatory element‐binding protein 2 (SREBP‐2) activity, improving hepatic lipid and glucose metabolism.^5^ Hepatic FXR activation was also found to lead to decreased activity of a closely related SREBP, SREBP‐1, which results in decreased lipogenesis and increased lipolysis.^6^

Evidence that these pharmacological off‐target effects of colesevelam may represent a class effect is suggested by the results of a short‐term, double‐blind, crossover trial demonstrating that cholestyramine, another BAS, may reduce serum glucose concentrations by 13% in non–insulin‐treated patients with diabetes.^7^

Colesevelam has been clinically studied in patients with type 2 diabetes mellitus (T2DM), with the commonest side effect being gastrointestinal disturbance, particularly constipation.^4^ In a systematic review,^4^ only few episodes of hypoglycemia were described, all of them being non‐severe. A 52‐week open‐label extension study on a population of 360 people with type 2 diabetes (on insulin, metformin, or sulfonylureas) showed that upon addition of colesevelam, 17 (3.3%) experienced mild‐to‐moderate hypoglycemia.^8^ From those subjects 11 were on insulin‐based, 5 on sulfonylurea‐based, and 1 on metformin‐based therapy. It is difficult to disentangle the overall effects of colesevelam from other antidiabetic agents, though, because of the paucity of studies comparing colesevelam to placebo in the absence of other glucose‐lowering agents.^4^

Regarding T1DM, a randomized double‐blind trial exploring potential benefits of colesevelam^9^ did not record any episodes of severe hypoglycemia. It is of note, however, that one of the exclusion criteria was the presence of previous unexplained severe hypoglycemia in the past 6 months,^9^ potentially making the inclusion of prone‐to‐hypoglycemia patients less likely. Another study showed no effect on postprandial glucose levels and concluded that colesevelam has no effect on peripheral insulin sensitivity or glucose absorption. However, it did increase whole‐body insulin sensitivity.^10^

Finally, a potential third mechanism involving the intestinal SGLT‐1 transporter is demonstrated in bariatric surgery studies. Bile diversion in gastric bypass modifies sodium‐glucose transport and postprandial glucose absorption, through the modulation of intestinal trafficking of endogenous sodium.^11^, ^12^ More specifically, following Roux‐en‐Y gastric bypass, glucose is only absorbed in the common intestinal limb, where ingested food meets bile. Conversely, glucose absorption is blunted in the bile‐deprived alimentary limb, while it is fully restored upon addition of bile in this segment.^12^ This finding demonstrates that in an intestinal segment where bile acid absorption does not take place, glucose absorption is also inhibited, introducing a potential correlation between bile acid sequestration and decreased intestinal glucose absorption. It has been proposed that the inhibition of SGLT‐1 sodium‐glucose transport in the alimentary limb could be induced by the deprivation of sodium load present in bile^11^; moreover, SGLT‐1 inhibition leads to increased GLP‐1 secretion.^13^, ^14^

## 6 CONCLUSION

We conclude that clinicians should be aware of hypoglycemia when combining sulphonylureas or insulin with BAS. Given the lack of SmPC (Summary of Product Characteristics) documentation for this side effect for bile acid sequestrants available for use in the UK (colesevelam and cholestyramine), this case highlights the need for clinicians to remain vigilant and report similar cases to the MHRA.

## CONFLICTS OF INTEREST

None.

## AUTHOR CONTRIBUTIONS

Dr Pavlou contributed to the writing of the manuscript, the designing of the figures and the graphical abstract as well as the interpretation of the insulin pump data. Dr Koutroukas contributed to the literature search and the writing of the manuscript, edited the figures and the graphical abstract, consented the patient, and completed the submission process. Dr Lissett conceived of the importance of the presented idea and provided critical feedback on the manuscript. Dr Smith supervised the work, edited the manuscript with useful suggestions about the phrasing and the structure, and provided critical feedback on the manuscript.

## ETHICAL APPROVAL

Hereby, I, Vaios Koutroukas, consciously assure that for this manuscript the following is fulfilled:

1. This material is the authors’ own original work, which has not been previously published elsewhere.
2. The paper is not currently being considered for publication elsewhere.
3. The paper reflects the authors’ own research and analysis in a truthful and complete manner.
4. The paper properly credits the meaningful contributions of co‐authors and co‐researchers.
5. All sources used are properly disclosed (correct citation).
6. All authors have been personally and actively involved in substantial work leading to the paper and will take public responsibility for its content.

## CONSENT

Patient consent form is signed by the patient.

## Data Availability

Data sharing is not applicable to this article as no new data were created or analyzed in this study.
